# Does a high body mass index remain a protective factor in hip fracture patients with hypertension and diabetes?

**DOI:** 10.1007/s40520-025-03010-x

**Published:** 2025-04-02

**Authors:** Zhening Guo, Weicheng Wu, Bo Lv, Yongtao Mao, Chang She, Wei Xu, Jun Gu, Liubing Li, Jie Pan

**Affiliations:** 1 Department of Pharmacy, The Second Affiliated Hospital of Soochow University, Suzhou, 215004 China; 2 Department of Orthopedics, The Second Affiliated Hospital of Soochow University, Suzhou, 215004 China; 3 State Key Laboratory of Radiation Medicine and Protection, Soochow University, Suzhou, 215123 China

**Keywords:** Hip fracture, BMI, HHS, Complication, Hypertension, Diabetes

## Abstract

**Background:**

Body mass index (BMI) was used to classify overweight or obesity. The obesity paradox was observed in elderly hip fracture patients. However, obesity has been implicated as one of the major risk factors for hypertension and diabetes. This study aims to determine whether a high body mass index (BMI) remains a protective factor in hip fracture patients with comorbid hypertension or diabetes, and to identify the optimal BMI threshold that best supports motor function recovery.

**Methods:**

This study included patients aged 65 years and older who have underwent hip fracture surgery. Harris Hip Score (HHS) was utilized to evaluate the functional recovery, the relationship between BMI and HHS was examined using both linear and generalized additive model (GAM). A threshold model was established with BMI of 24 kg/m^2^ and the difference between the threshold model and the GAM was compared utilizing the likelihood ratio test (LRT).

**Results:**

A total of 213 patients were enrolled in the study. A nonlinear relationship was identified between BMI and HHS in patients with either hypertension or diabetes and the HHS demonstrated a significant downward trend with increasing BMI. The LRT revealed no significant difference between the threshold effect model with a BMI value of 24 kg/m^2^ and the GAM.

**Conclusions:**

This study reveals that the protective effect of high BMI on postoperative motor function in hip fracture patients is significantly modulated by comorbidities. We recommend modulating the BMI to approximately 24 kg/m^2^ for elderly patients with hip fractures and comorbid conditions such as hypertension and diabetes.

## Introduction

In recent years, with global population aging and lifestyle changes, the incidence of hypertension and diabetes has risen significantly, becoming major chronic diseases affecting global health. In 2019, over 1.2 billion people worldwide were affected by high blood pressure, and 463 million were diagnosed with diabetes [1]. Numerous studies have demonstrated the associations between obesity and the development and poor prognosis of cardiovascular diseases, hypertension, and non-insulin-dependent diabetes [2]. The costs of preventing and treating these diseases are increasing significantly, contributing to the growing concern of obesity as a global public health issue [3]. Excess weight is responsible for approximately 14% of male deaths and 20% of female deaths [4, 5].

However, in certain diseases, overweight and obesity have been observed as a prognostic protective factor, commonly referred to as the “obesity paradox”. In 1999, Fleischmann discovered that overweight hemodialysis patients had significantly greater one-year survival rates than patients with normal weight [6]. Furthermore, in 2002, Gruberg et al. reported that obese patients with coronary artery disease had lower mortality and complication rates than normal-weight patients with the same condition. This groundbreaking study introduced the concept of the “obesity paradox” [7], highlighting that individuals with a higher body mass index (BMI) tend to experience a better prognosis.

Hip fracture is considered “the last fracture of the elderly”. Its incidence increases with age, and it is among the top ten causes of disability. The number of hip fractures worldwide is expected to increase from 1.26 million in 1990 to 4.5 million in 2050 [8]. Most patients who suffer from hip fracture require the use of a walker for a year afterward [9], as well as prolonged hospitalization and rehabilitation, thus heavily burdening healthcare [8]. Given its severe implications, hip fracture is recognized as a critical type of osteoporotic fracture [8, 10]. A particular Swedish cohort study involving 17,756 hip fracture patients revealed a 30–35% lower one-year mortality rate among overweight individuals compared to their normal-weight counterparts, indicating the existence of the “obesity paradox” in hip fracture patients [11]. The underlying mechanisms of this paradox remain elusive, though they may be associated with the protective effects of higher bone mineral density (BMD) found in overweight individuals [12] or the potential of their adipose tissue to act as a metabolic reserve, warding off rapid muscle deterioration [13]. Furthermore, research conducted by Karin et al. indicated that the “obesity paradox” is more evident in patients older than 65 years, suggesting that weight loss recommendations for overweight patients to improve clinical outcomes might not be universally applicable. Hence, the advisability of weight loss for overweight or obese hip fracture patients to improved prognosis post-hip fracture requires careful consideration, particularly among the elderly patients [11, 14].

With advancing age, both bone fragility and the propensity for falls increase, disproportionately affecting the elderly. Beside, the incidence of obesity associated diseases, such as hypertension, diabetes, coronary artery disease, atrial fibrillation, and heart failure, is also related to age [15]. These comorbidities are commonly observed in elderly hip fracture patients contributing to a higher mortality rate [16, 17]. Obese individuals have a 3.5 times higher risk of developing hypertension, of which 60% can be ascribed to increased fat stores [18]. Hypertension, in turn, serves as an independent risk factor for readmission in elderly hip fracture patients [18]. Obesity plays a significant role in the development of diabetes and contributes to its exponential increase [19]. Diabetic patients face a greater risk of fragility fractures, particularly hip fractures, compared to nondiabetic patients [20]. Moreover, diabetic patients experience a substantially higher absolute risk of mortality after fracture [21], while their postoperative recovery of motor function is also affected [22, 23].

Weight management plays a crucial role in decreasing the occurrence of obesity-related comorbidities. However, the “obesity paradox” phenomenon suggests that a higher BMI might offer protection for individuals with hip fractures and chronic diseases. Thus, in view of this contradictory phenomenon, we propose a scientific hypothesis that the obesity paradox is not simply applicable in elderly hip fracture patients with obesity associated diseases. There is a cut point in BMI, and this optimal BMI value can promote the best recovery of postoperative motor function after hip fractures surgery. This study aimed to retrospectively analyze the influence of BMI on motor function recovery one year after surgery in this specific demographic. By exploring the correlation between BMI and postoperative motor function improvement, our findings will contribute to weight management strategies for older patients with obesity-related comorbidities who suffer hip fractures surgery.

## Materials and methods

### Study population

This study conducted a retrospective analysis of patients who underwent hip fracture surgery between January 2021 and June 2022. Eligible patients were all aged 65 years or older and underwent surgical procedures such as hip replacement and fixation. We excluded individuals with fractures due to pathological causes such as malignant tumors, ischemic necrosis, those with a history of hip joint surgery, and cases of high-energy trauma, as well as patients with incomplete data. Participants were categorized into four groups based on their medical history: hypertension, diabetes, non-hypertension and non-diabetes. Hypertension was defined as self-reported hypertension under treatment and/or a clinical blood pressure reading with systolic pressure ≥ 140mmHg and/or diastolic pressure ≥ 90mmHg. Diabetes was identified based on self-reported diabetes under treatment with hypoglycemic drugs or insulin, or if the patient’s fasting blood glucose level at admission was ≥ 7.0 mmol/L or the 2-hour post-glucose tolerance test (OGTT) blood glucose level was ≥ 11.1 mmol/L. The hospital’s institutional review committee granted approval for this study. In clinical settings and epidemiological research, body mass index (BMI) is commonly used to classify overweight or obesity [24]. The World Health Organization defines a BMI ≥ 25 kg/m^2^ as overweight and a BMI ≥ 30 kg/m^2^ as obese. For the Chinese population, we often further categorize body mass index as underweight (BMI ≤ 18.4 kg/m^2^), normal weight (18.5 kg/m^2^ ≤ BMI ≤ 23.9 kg/m^2^), overweight (24.0 kg/m^2^ ≤ BMI ≤ 27.9 kg/m^2^) and obese (BMI ≥ 28.0 kg/m^2^) [25]. The study was approved by the hospital’s Medical Ethics Committee (JD-HG-2023-07).

### Data collection

For each patient, we meticulously reviewed their medical history and compiled demographic and clinical-pathological data, such as gender, age, type of fracture, and surgical method employed. Patient height and weight were measured following standard procedures, and their body mass index (BMI) was calculated using the formula: weight (kg) / height squared (m [2]). To evaluate mor functional recovery one year after surgery, we utilized the Harris Hip Score (HHS). The HHS is a comprehensive assessment tool for hip joint function, with a maximum score of 100. It encompasses various subscales, with higher weights assigned to activities of daily living and gait (47 points), and pain (44 points), and lower weights to absence of deformity (4 points) and range of motion (5 points). This scoring system has been previously validated for assessing patient outcomes following hip surgery [26].

### Statistical analysis

Statistical analyses in this study were performed using R software (version 4.0.2). We presented quantitative variables as means ± standard deviations, and categorical variables as counts and percentages. Analysis of variance (ANOVA) was applied to determine significant differences in anthropometric and clinical indicators across the various groups (hypertension, non-hypertension, diabetes, and non-diabetes). Adjusting for factors such as gender, age, fracture type, and surgical method, we utilized both a linear model and a generalized additive model (GAM) with a plate regression spline (from the mgcv 1.8–41 package) to elucidate the relationship between BMI and HHS. A threshold model was used for further testing, and a likelihood ratio test was used to compare the threshold model and its differences with the GAM model. *P* < 0.05 was considered to indicate statistical significance.

## Results

### Clinicopathological characteristics

Within the primary cohort of 266 patients, after excluding those who were lost to follow-up, deceased, or had incomplete data, a total of 213 patients met the inclusion criteria and were enrolled in the study. This group comprised 69 males (32.4%) and 144 females (67.6%). In terms of BMI categories, there were 27 underweight patients (12.7%), 114 with normal weight (53.5%), 48 classified as overweight (22.5%), and 24 as obese (11.3%). The cohort included 124 patients diagnosed with hypertension (58.2%) and 70 with diabetes (30.3%) (Table 1).

**Table 1:** Clinical characteristics of hip fracture patients

Variable	Under Weight	Normal	Over Weight	Obesity	*p*
*n*	27	114	48	24	
Age	81.07 (8.85)	77.62 (7.76)	76.31 (6.04)	73.38 (7.26)	0.003
Gender					
Female	22 (81.5)	74 (64.9)	32 (66.7)	16 (66.7)	0.427
Male	5 (18.5)	40 (35.1)	16 (33.3)	8 (33.3)	
Fracture type					
FNF	19 (70.4)	71 (62.3)	29 (60.4)	10 (41.7)	0.186
ITF	8 (29.6)	43 (37.7)	19 (39.6)	14 (58.3)	
Surgery type					
Replacement	17 (63.0)	68 (59.6)	30 (62.5)	11 (45.8)	0.540
Fixation	10 (37.0)	46 (40.4)	18 (37.5)	13 (54.2)	
Hypertension					
No	18 (66.7)	45 (39.5)	15 (31.2)	11 (45.8)	0.024
Yes	9 (33.3)	69 (60.5)	33 (68.8)	13 (54.2)	
Diabetes					
No	20 (74.1)	75 (65.8)	32 (66.7)	16 (66.7)	0.876
Yes	7 (25.9)	39 (34.2)	16 (33.3)	8 (33.3)	
HHS	69.07 (6.01)	79.05 (8.41)	81.58 (5.96)	81.00 (8.98)	< 0.001

Abbreviations: BMI, body mass index; ITF, Intertrochanteric fracture; FNF, femoral neck fracture

### Linear and Non-Linear Relationship Between BMI and HHS

Following adjustments for gender, age, surgical method, and fracture type, we performed a linear trend analysis to investigate the relationship between BMI and HHS. As indicated in Table 2, the effect value of BMI in patients with hypertension and diabetes showed an initial increase followed by a decrease. This pattern does not represent a significant linear trend, suggesting the possibility of a nonlinear effect.

**Table 2:** The linear regression model to evaluate relationship between BMI and HHS

Model		Effect [95% CI]	*P* for trend
Overall			
	Under Weight (< 18.5)	-9.618 [-12.775, -6.46]	< 0.001
	Normal(18.5–24)	Ref	
	Over Weight(24–28)	2.203 [-0.254, 4.659]	
	Obesity(> 28)	0.605 [-2.661, 3.87]	
Hypertensive			
	Under Weight (< 18.5)	-8.488 [-14.145, -2.832]	0.298
	Normal(18.5–24)	Ref	
	Over Weight(24–28)	1.042 [-2.272, 4.356]	
	Obesity(> 28)	-2.098 [-6.845, 2.649]	
Non-Hypertensive			
	Under Weight (< 18.5)	-10.325 [-13.855, -6.794]	< 0.001
	Normal(18.5–24)	Ref	
	Over Weight(24–28)	4.359 [0.709, 8.01]	
	Obesity(> 28)	4.209 [-0.249, 8.667]	
Diabetes			
	Under Weight (< 18.5)	-4.823 [-11.033, 1.387]	0.614
	Normal(18.5–24)	Ref	
	Over Weight(24–28)	1.247 [-3.057, 5.551]	
	Obesity(> 28)	-2.12 [-7.756, 3.516]	
Non-Diabetes			
	Under Weight (< 18.5)	-11.346 [-15.015, -7.676]	< 0.001
	Normal(18.5–24)	Ref	
	Over Weight(24–28)	2.641 [-0.345, 5.628]	
	Obesity(> 28)	1.533 [-2.444, 5.51]	

**Fig. 1:**
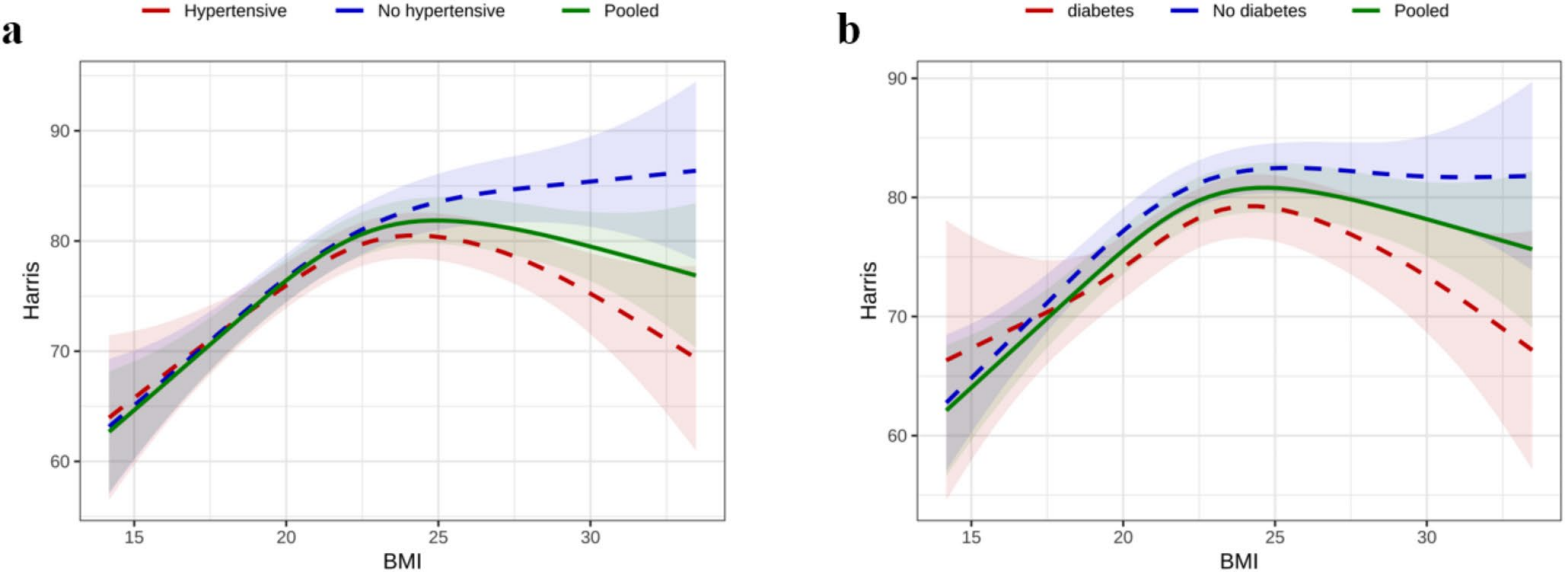
The non-linear relationship between BMI and HHS in hypertension(**a**) and diabetes(**b**) Abbreviations: BMI, body mass index; HHS, Harris Hip Score

To assess the potential non-linear relationship between BMI and HHS, we employed a generalized additive model (GAM). The analysis revealed, through a smooth curve (adjusted for gender, age, fracture type, and surgery type), that BMI indeed exhibited a non-linear relationship with HHS (Fig. 1). Notably, in patients with either hypertension or diabetes, the HHS demonstrated a significant downward trend at higher BMI levels

### Analysis of the threshold effect model

**Fig. 2:**
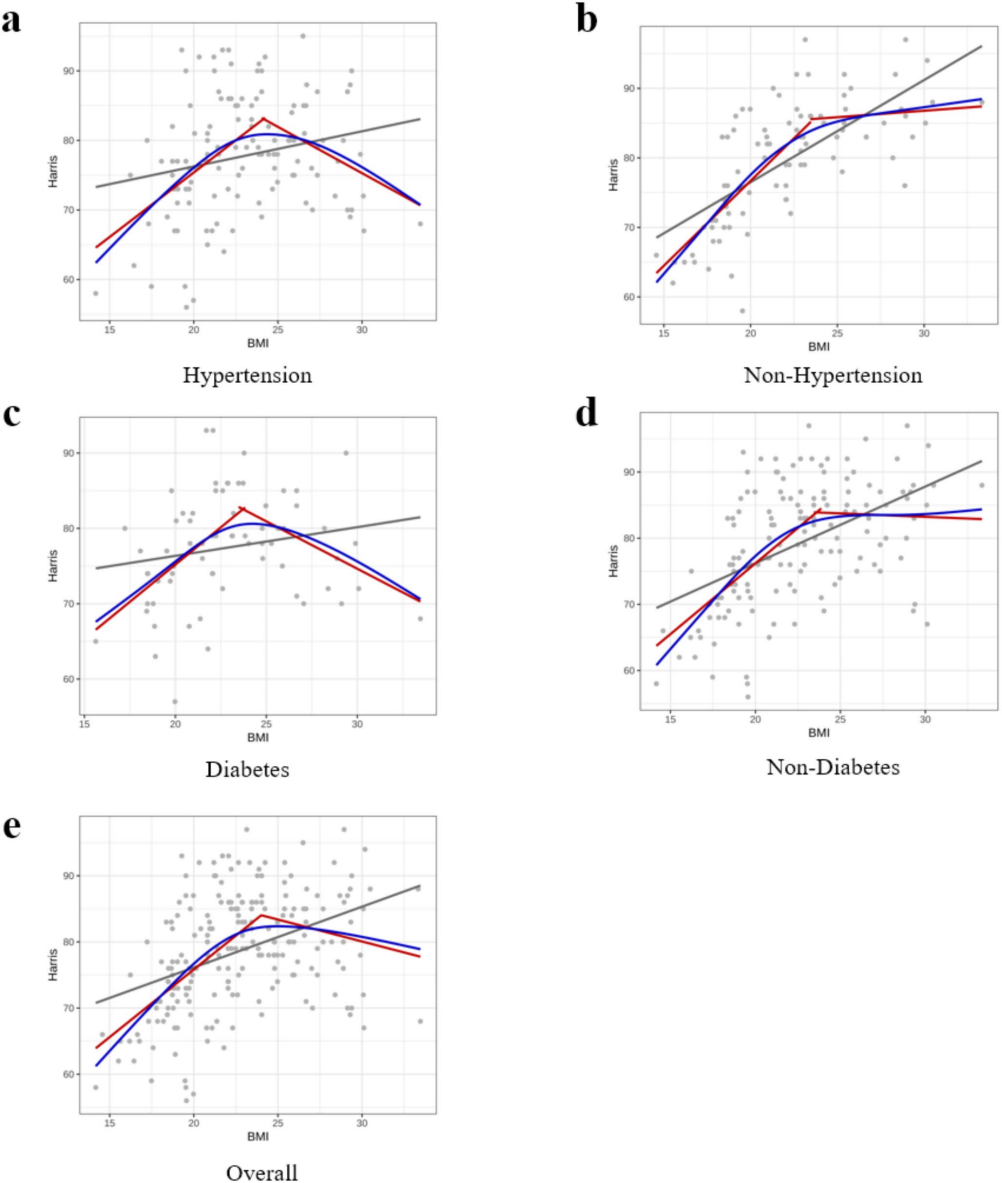
Linear model, GAM curve, and threshold model of the relationship between BMI and Harris with hypertension(**a**), non-hypertension(**b**), diabetes(**c**), non-diabetes(**d**) and overall(**e**) Abbreviations: GAM, generalized additive model

Establishing a threshold effect model with a BMI value of 24 kg/m^2^, the likelihood ratio test (LRT) revealed no significant difference between the threshold effect model and the generalized additive model (GAM), indicating that the threshold effect model is a viable alternative to the smooth curve generated by the GAM (Table 3). This finding suggested that BMI exerts a significant threshold effect on the Harris Hip Score (HHS) across the entire study population. Specifically, BMI has a positive impact on HHS up to the point of overweight status, beyond which the effect turns negative. However, for healthy individuals (those without underlying conditions), this negative effect is not pronounced, even at an overweight BMI. In contrast, individuals with underlying diseases exhibit a reversal effect after reaching an overweight BMI, highlighting that while healthy individuals experience a saturation effect at higher BMI levels, those with underlying diseases encounter an adverse effect (Fig. 2).

**Table 3:** The threshold model of BMI on Harris

Model		Slope [95% CI]
Overall		
Ordinary model		
	BMI	0.796 [0.517, 1.075]
Threshold model		
	BMI (< threshold)	2.009 [1.566, 2.451]
	BMI (> threshold)	-0.861 [-1.421,-0.301]
	*P* for threshold	< 0.001
	*P* value in LRT	0.9921
Hypertensive		
Ordinary model		
	BMI	0.392 [-0.009, 0.794]
Threshold model		
	BMI (< threshold)	1.750 [1.069, 2.431]
	BMI (> threshold)	-1.315 [-2.132,-0.497]
	*P* for threshold	< 0.001
	*P* value in LRT	0.9993
No Hypertensive		
Model without Threshold		
	BMI	1.357 [1.003, 1.711]
Threshold model		
	BMI (< threshold)	2.369 [1.862, 2.876]
	BMI (> threshold)	-0.291 [-1.024, 0.441]
	*P* for threshold	< 0.001
	*P* value in LRT	0.9993
Diabetes		
Model without Threshold		
	BMI	0.345 [-0.146, 0.837]
Threshold model		
	BMI (< threshold)	1.860 [1.063, 2.658]
	BMI (> threshold)	-1.433 [-2.348, -0.518]
	*P* for threshold	< 0.001
	*P* value in LRT	0.9997
No Diabetes		
Model without Threshold		
	BMI	0.9571 [0.624, 1.290]
Threshold model		
	BMI (< threshold)	2.048 [1.523, 2.573]
	BMI (> threshold)	-0.648 [-1.351, 0.056]
	*P* for threshold	< 0.001
	*P* value in LRT	0.9403

Abbreviations: BMI, body mass index

## Discussion

This study revealed that the protective effect of high BMI on postoperative motor function in hip fracture patients is significantly modulated by comorbidities, challenging the universal applicability of the “obesity paradox” in this demographic. Hypertension and diabetes, prevalent complications in elderly hip fracture patients, considerably influence prognosis [27, 28]. We investigated the impact of varying BMI levels on functional recovery in hip fracture patients, with and without hypertension and diabetes, one year post surgery. A novel nonlinear GAM analysis was employed for statistical evaluation. After controlling for gender, age, fracture type, surgical method, and other confounding factors, our findings suggest that the “obesity paradox” holds true for hip fracture patients without complications like hypertension or diabetes. Consistent with prior research [29], higher BMI values correlate with improved post-surgical motor function in these patients. Conversely, in patients with hypertension and diabetes, a nonlinear relationship between BMI and the Harris Hip Score (HHS) was observed. Particularly, excessively high BMI levels led to a marked decline in HHS. The likelihood ratio test (LRT) confirmed that the threshold effect model, established at a BMI of 24 kg/m^2^, aligns closely with the GAM results. These findings indicate a negative correlation between BMI and HHS in hip fracture patients with comorbidities, suggesting that higher BMI adversely affects functional recovery. Therefore, for elderly hip fracture patients with hypertension and diabetes, maintaining a BMI close to 24 kg/m^2^ is advisable for optimal post-discharge functional recovery.

The beneficial aspects of overweight or obesity in the context of normal aging remain ambiguous, and the underlying mechanisms of the “obesity paradox” are not fully understood. Research indicates that following a hip fracture, patients may experience stress reactions leading to metabolic disorders and prolonged metabolic breakdown [30]. In such scenarios, a higher BMI may act as a metabolic reserve, helping to prevent acute muscle loss and weakness, thereby facilitating the recovery of motor function [13]. A 2017 review highlighted that most evidence supports a correlation between higher BMI and increased bone mineral density, characterized by lower bone turnover and greater bone strength [31]. This relationship might explain the “obesity paradox” observed in patients with hip fractures.

For patients with hypertension and diabetes, the explanations underpinning the “obesity paradox” may not apply entirely. The link between obesity and hypertension is well-established. Fahad’s study notes that hypertensive patients generally have a higher BMI than patients without hypertension. Moreover, the risk of developing hypertension escalates with increasing BMI, particularly beyond a BMI of 23 kg/m^2^ [32]. Seravalle’s research suggests that the mechanisms of obesity and obesity-related hypertension are complex and sometimes interdependent. The main role, other than genetic and environmental factors, is due to the sympathetic nervous system, to renal and adrenal function, to the endothelium, to the adipokines, and to the insulin resistance [18]. Obese individuals with hypertension are at an increased risk of additional complications detrimental to prognosis, such as cardiovascular disease, hyperuricemia, kidney injury, left ventricular hypertrophy, reduced cardiac function, and cognitive impairment [33, 34]. This increased disease burden may contribute to poorer functional recovery in hip fracture patients with hypertension at higher BMI levels. Fantin’s study indicates that weight loss can positively affect blood pressure control [35]. Stable blood pressure is even predictive of fracture rates one year after surgery, underscoring the importance of managing both blood pressure and BMI for better prognostic outcomes in these patients [28].

Numerous studies have established that obesity is a significant risk factor for diabetes, with a notably higher prevalence of diabetes among obese individuals compared to their non-obese counterparts. Diabetic patients typically have higher BMI values [23, 36]. The interrelation between obesity and diabetes is substantial. Diabetic patients face an increased risk of hip fractures, with factors like hypercalciuria, impaired renal function, insulin deficiency, and microvascular complications accelerating bone loss. This can adversely affect motor function recovery post-fracture and lead to elevated mortality rates [22, 37]. Although diabetic patients often have higher bone mineral density than non-diabetics, their bones are more prone to fragility. The mechanisms underlying skeletal fragility in diabetes are not completely understood, but are multifactorial and likely include effects of obesity, hyperglycaemia, oxidative stress, and accumulation of advanced glycation end products, leading to altered bone metabolism, structure, and strength [38]. These may be the reasons why the motor functional recovery of diabetes patients with high BMI is not ideal.

It’s crucial to focus on the elderly population, particularly in the context of hip fractures. While BMI is a widely used anthropometric tool for assessing the nutritional status of the elderly and sometimes for managing malnutrition, a high BMI should not be automatically equated with good nutrition. Specifically, for hip fracture patients with hypertension and diabetes, the “obesity paradox” does not hold true. Traditional BMI assessments have their limitations in this regard. For healthy hip fracture patients, appropriate nutritional supplementation to increase BMI values may be beneficial. Nutritional therapy has been shown to enhance postoperative albumin and total protein levels, shorten bed rest duration, expedite recovery, reduce complications, and improve quality of life in elderly patients with hip fractures. However, for those with hypertension and diabetes, it’s advisable to maintain a BMI close to 24 kg/m^2^– neither too low nor too high. In obese or overweight individuals, nutritional interventions can effectively optimize body composition and BMI [39]. Nonetheless, further research with larger populations and more extensive data is necessary for validation. The BMI target values identified in this study can inform more precise, personalized nutritional treatment plans for elderly hip fracture patients with obesity-related comorbidities, significantly impacting their quality of life, motor function, disability reduction, and mortality.

This study has several limitations. First, the sample size was relatively small, which may introduce bias into the results and limit the generalizability of the findings. Second, while BMI is used as a proxy for obesity, it does not account for body composition. Future research will integrate of precise metrics, including waist circumference and waist-to-hip ratio, to investigate in greater depth the complications associated with BMI that impact the prognosis of patients with hip fractures. Third, due to incomplete data availability regarding the duration of treatment, types of medications, and dosage regimens for all patients, these variables were not included in the current analysis.

## Conclusion

In our study of patients older than 65 years with hip fractures, we found that the “obesity paradox” can’t apply to those concomitant hypertension and diabetes patients. A non-linear relationship was observed between BMI values and the Harris Hip Scores one year after surgery. Specifically, for individuals with a BMI of 24 kg/m^2^ or higher, there was a negative correlation with HHS. For hip fracture patients over 65 years of age, we recommend enhancing nutritional support, such as increasing the intake of high-quality protein and appropriately using oral nutritional supplements (ONS) [40], to promote weight gain, which can aid in motor function recovery. However, for patients with comorbid hypertension and diabetes mellitus, we advise maintaining a BMI below 24 kg/m^2^ for optimal outcomes. However, to validate and expand upon these findings, further multicenter, prospective, and randomized controlled studies are necessary.

## Data Availability

No datasets were generated or analysed during the current study. Data sharing is not applicable.
